# Adipose Tissue-Derived Biomarkers of Intestinal Barrier Functions for the Characterization of Diarrhoea-Predominant IBS

**DOI:** 10.1155/2018/1827937

**Published:** 2018-11-28

**Authors:** Francesco Russo, Guglielmina Chimienti, Giuseppe Riezzo, Michele Linsalata, Benedetta D'Attoma, Caterina Clemente, Antonella Orlando

**Affiliations:** ^1^Laboratory of Nutritional Pathophysiology, National Institute of Gastroenterology “S. de Bellis”, Research Hospital, Castellana Grotte Bari, Italy; ^2^Department of Biosciences, Biotechnology and Biopharmaceutics, University of Bari, Bari, Italy

## Abstract

**Background:**

Alterations of the small-intestinal permeability (s-IP) might play an essential role in a subgroup of diarrhoea-predominant IBS (D-IBS) patients.

**Goals:**

(*a*) To analyse in D-IBS patients the symptom profile in relation to the altered (+) or not (−) s-IP using the Gastrointestinal Symptom Rating Scale (GSRS). (*b*) To assess the circulating levels of the adipokines IL-6, IL-8, TNF-*α*, leptin, and adiponectin, along with LPS, TLR-4, neurotensin, and brain-derived neurotrophic factor (BDNF). The frequency distribution of SNPs at the loci for the investigated molecules and leptin receptor was evaluated.

**Study:**

The study included 34 D-IBS patients and 17 healthy controls (HC). s-IP permeability was assayed by high-performance liquid chromatography determination in the urine of the lactulose to mannitol ratio. Concentrations of IL-6, IL-8, TNF-*α*, LPS, TLR-4, leptin, adiponectin, neurotensin, and BDNF were assayed by ELISA. Screening of genetic variants was done employing the restriction fragment length polymorphism-polymerase chain reaction method.

**Results:**

D-IBS(−) patients had a significantly higher GSRS cluster pain and diarrhoea profile than D-IBS(+) ones. Significant correlations were found between the symptoms clusters and immune activation and inflammation markers. The levels of adipo(cyto)kines in D-IBS(+) patients were higher than those of controls, and IL-6 levels correlated with those of LPS. Leptin and BDNF were significantly higher, and neurotensin levels were significantly lower in D-IBS(+) than in controls. No differences were found in the frequency distribution of genotypes among the study groups.

**Conclusions:**

Results from this study could be of some help in the characterization of the D-IBS and highlight the contribution of an altered intestinal barrier in the pathogenesis of this syndrome. Besides, a role could be ascribed to molecules secreted by the visceral adipose tissue that can impact on barrier functions.

## 1. Introduction

Irritable bowel syndrome (IBS) is one of the most common functional gastrointestinal disorders (FGIDs), with a prevalence ranging from 9% to 23% of the worldwide population [1]. It has a complex pathology characterised by a continuum of symptoms (abdominal pain, discomfort, or both in association with altered bowel habits, irregular stool form and passage, and bloating), often overlapping with other FGIDs [2] and organic diseases characterised by an IBS-like symptom profile (i.e., small intestinal bacterial overgrowth (SIBO), celiac disease, gluten sensitivity, fermentable oligosaccharide, disaccharide, monosaccharide, and polyol (FODMAP) intolerance, lactose intolerance, and nickel allergic mucositis) [3]. Once major organic gastrointestinal (GI) disorders are excluded, a diagnosis labelled as “IBS” should be taken into account, based on the symptom phenotype and stool characteristics [4, 5]. However, in a specific subgroup of patients suffering from diarrhoea-predominant IBS (D-IBS), alterations in the intestinal barrier have been demonstrated, narrowing the spectrum of IBS as a true functional disease [6, 7]. The intestinal barrier acts as a sophisticated anatomical and functional structure lying between the strictly regulated intestinal milieu and the external environment.

On the one hand, this barrier protects the host from the invasion of pathogenic microorganisms and toxins. On the other hand, it allows the absorption of nutrients and fluids [8]. A dysfunctional gut barrier leads to variations of small intestinal permeability (s-IP) and could be the origin or the consequence of the persistent, low-grade immune activation characterising the abovementioned D-IBS subtype and, with greater severity, other inflammatory GI diseases, such as inflammatory bowel disease (IBD) and celiac disease (CD) [6, 9]. The intestinal barrier functions are affected by the enteric nervous system (ENS), both directly through regulation of tight junctions via neurotransmitters and neurotrophic factors [10] and indirectly through neuroimmune modulation during inflammation [11].

The study of mediators involved in the regulation and maintenance of epithelial barrier integrity could add new insights in the search for reproducible biomarkers of the pathophysiology of IBS [12]. An interplay between visceral adipose tissue, located close to the GI tract, and intestinal permeability has been demonstrated [13]. Adipose tissue is the most extensive endocrine organ, being the source of active molecules able to affect both physiological and pathological processes and a source of neurological mediators involved in the cross-talk between the ENS and central nervous system (CNS) [14].

In this framework, the overall objective of our study was to investigate the involvement of adipose tissue-derived hormones, immune- and neuromodulatory factors in the alterations of the s-IP in D-IBS patients categorised as having normal [D-IBS(−)] or increased s-IP [D-IBS(+)] by using the lactulose/mannitol (La/Ma) ratio and healthy subjects. In particular, the aims of the study were to (a) analyse the symptom profile in D-IBS patients using a validated questionnaire, such as the Gastrointestinal Symptom Rating Scale (GSRS), in relation to whether or not there are alterations in s-IP [15] and (b) assess the circulating levels of the adipo(cyto)kines interleukin 6 (IL-6), interleukin 8 (IL-8), and tumor necrosis factor alpha (TNF-*α*) that play a role as mediators of inflammation and immune activation [16], along with circulating levels of lipopolysaccharide (LPS) and toll-like receptor 4 (TLR-4), as markers of endotoxemia; leptin and adiponectin as mediators of intestinal inflammation [17]; neurotensin, with its role in preserving gut barrier integrity [18]; and the metabotropic brain-derived neurotrophic factor (BDNF), a neuroendocrine factor expressed in adipose tissue [19]. Lastly, the familial aggregation suggests a genetic component as having a role in increasing the risk of developing the pathology, although the functional role of allelic variants in IBS is still an open field [20]. Looking for a genetic make-up related to alteration of intestinal permeability, we evaluated the frequency distribution of single-nucleotide polymorphisms (SNPs) at the loci for the investigated molecules and leptin receptor in patients and controls. Namely, we considered the upstream variants rs1800795 in the IL-6 gene, rs4073 in the CXCL8 (IL-8) gene, and rs1800629 in the TNF-*α* gene; rs7799039 in the gene coding for leptin; rs1800832 in the 5′ UTR of the neurotensin gene; rs1137101 in the coding sequence of the leptin receptor gene; rs6265 in the coding sequence of the BDNF gene; the intron variants rs2227303 in the IL-8 gene; and rs1501299 at the ADIPOQ locus.

## 2. Patients and Methods

### 2.1. Study Participants

Patients suffering from diarrhoea-predominant IBS according to Rome III criteria, from June 2013 to February 2015, were recruited in this prospective case-control study from among the outpatients of the National Institute of Gastroenterology “S. de Bellis,” Research Hospital, Castellana Grotte (Bari), Italy.

All the patients underwent a validated questionnaire for GI symptoms (see below and dedicated section), physical examination, whole blood count, liver function tests, stool routine, faecal occult blood test, stool culture, stool examination for parasites, C-reactive protein, thyroid function test, gastroscopy, and colonoscopy to avoid the enrolment of patients with organic diseases.

The inclusion criteria were as follows: (a) age more than 18 years; a symptom profile resembling D-IBS with a stool pattern, as described according to Schmulson et al. [21]; (b) active symptoms for at least 2 weeks; (c) a minimum average of 3.0 on the seven-point Likert scale of the GSRS composite symptom score [15]; (d) a diet without any restriction on eating and drinking (in particular, no previous period of gluten-free diet (GFD) before examination); (e) as gluten-sensitive diarrhoea without celiac disease (CD) is a clinical entity that has been observed in IBS patients positive for HLA-DQ2 or HLA-DQ8 [22], only the HLA-DQ2/HLADQ8-negative/negative D-IBS patients were considered for this study; (f) age, BMI, smoking, alcohol intake, and use of medication was accurately checked in order to obtain a group of D-IBS as homogeneous as possible.

Exclusion criteria included postinfectious IBS, hepatic, renal, or cardiovascular disease; constipation; metabolic and endocrine disorders; history of SSRIs and other antidepressant therapy; fever; intense physical activity; previous abdominal surgery; history of malignancy; secondary causes of intestinal atrophy; pregnancy; and lactose intolerance or giardiasis. Besides, patients did not have to consume medication for the treatment of IBS for two weeks before evaluation, antibiotic therapy or probiotic agents, and other drugs known to cause abdominal pain. The exclusion of CD was performed following the international guidelines and published data. Serologic testing, with a combination of tissue transglutaminase (tTG) and anti-endomysium antibodies (EMA), was used.

The reasons for study discontinuation were recorded in the case report form and could include death, adverse event (specified), ineligibility to continue the study, lost to follow-up, withdrew consent, and other (including the administrative closure of trial).

Healthy individuals were enrolled from among the administrative staff of our institute as controls (HC). They denied having metabolic, endocrine, or immunological diseases, dyspepsia, or other GI diseases and did not take any medication. Information on the health status of participants was obtained by an interview on the current diet, lifestyle, medical history, and a physical examination. As criteria of admission, EMA and tTG had to be negative. Besides, metabolic parameters (blood glucose, HbA1c, lipid profile, body weight, and blood pressure) had to be within the normal range of values. The absence of major psychiatric disorders, cancer, and pregnancy was also inclusion criteria. All the women, both patients and controls, were examined during the follicular phase of the menstrual cycle.

All the participants (both D-IBS and HC) were subjected to all the scheduled analyses. The D-IBS patients were categorised as having normal [D-IBS(−)] or increased s-IP [D-IBS(+)], by using the sugar absorption tests [23].

All the subjects were compliant and were willing to participate in the study. Informed consent was obtained from all the patients and healthy participants for blood testing and clinical data collection. This study was approved by the local Scientific and Ethics Committees of IRCCS “Saverio de Bellis,” Castellana Grotte, Bari, Italy, and it was part of a registered research on https://clinicaltrials.gov/ct2/show/NCT01574209).

### 2.2. Symptom Assessment

Patients were evaluated with the Gastrointestinal Symptom Rating Scale (GSRS), a validated questionnaire for GI symptoms [15]. GSRS utilises a seven-level Likert scale (1–7), depending on intensity and frequency of GI symptoms experienced during the previous week. A higher score indicates mainly inconvenient symptoms. Combination scores among the questions were calculated for the following domains: “*abdominal pain*” (pain referred as epigastric, colic, continuous, or indefinite pain, gastric hunger pains, and nausea; max. score: 42), “*indigestion syndrome*” (postprandial fullness, early satiety, borborygmi, bloating, eructation/belching, and increased flatus; max. score: 42), and “*diarrhoea syndrome*” (increased frequency of evacuation, loose stools, and urgent need to defecate; max. score: 21).

### 2.3. Analytical Measurements

All the analytical measurements were performed at the time of enrolment using blind-coded samples (no name or personal identifiers). Peripheral venous blood samples were obtained from participants in the study in the fasting state at least twelve hours after the last meal. After allowing clotting for at least 30 min, the samples were centrifuged at 1600 ×*g* for 15 min. Besides, a whole-blood sample was obtained, and DNA was extracted.

Serum leptin, adiponectin, neurotensin, BDNF, and plasma IL-6, IL-8, TNF-*α*, LPS, and TLR-4 concentrations were measured in duplicate using commercially available sandwich enzyme-linked immunosorbent assay kits: Human Leptin ELISA—Diagnostic Biochem, Canada Inc., Ontario, Canada; Human Adiponectin ELISA, High Sensitivity—Biovendor GmbH, Heidelberg, Germany; ELISA kit for neurotensin—Cloud-Clone Corp., TX, USA; Human Free BDNF Elisa kit—R&D Systems Inc., MN, USA); Human IL-6 Quantikine ELISA, Human IL-8 Quantikine ELISA, and Human TNF-alpha Quantikine ELISA kits—BD Biosciences, Milan Italy; Lipopolysaccharide (LPS) ELISA kit—Cloud-Clone Corp., Katy, TX, USA; Human Toll-Like Receptor 4 (TLR-4) ELISA kit—Cloud-Clone Corp., Katy, TX, USA.

### 2.4. Sugar Absorption Tests

For the evaluation of intestinal permeability, a test solution was prepared containing 40 g sucrose (Su), 10 g lactulose (La), and 5 g mannitol (Ma) dissolved in 100 ml of water.

The participants drank the test solution in the morning after an overnight fast and all urine samples were collected for the subsequent five hours. Urine samples were stored at −80°C until analysis. The detection and measurement of the three sugar probes, Su, La, and Ma, in urine were performed by chromatographic analysis as described previously by our group [23]. Briefly, high-performance anion exchange chromatography coupled with pulsed amperometric detection was performed on a Dionex Model ICS-5000 with a gold working electrode and a 25 *μ*l peek sample loop (Dionex Corp., Sunnyvale, California, USA).

The carbohydrate separation was performed using a CarboPac PA-10 pellicular anion-exchange resin connected to a CarboPac PA-10 guard column (Thermofisher Scientific, Waltham, Massachusetts, USA) at 30°C. The samples were eluted with 50 mmol/l NaOH at a flow rate of 1 ml/min. The percentages of ingested Su (%Su) together with those of La (%La) and Ma (%Ma) in urine were evaluated, and the La/Ma ratio was calculated for each sample. Based on data from controls in our laboratory, La/Ma ≥ 0.035 is indicative of increased intestinal permeability [23].

### 2.5. Genotyping

Screening of genetic variants at the loci considered for this study was done employing the restriction fragment length polymorphism-polymerase chain reaction (RFLP-PCR) method.

The primer sequences, reaction conditions for genotyping assays, and references are reported in the supplementary data section (available here).

### 2.6. Statistical Analysis

All results are expressed as mean ± SEM or median and range in the case of continuous or discrete variables, respectively. The rank sum test or the Kruskal-Wallis with Dunn's posttest were used where appropriate. Relationships between parameters were assessed using the Spearman correlation coefficient. The *χ* ^2^ test was used to investigate the genotype frequency distributions. All the differences were considered significant at a 5% level. A specific statistical package for exact nonparametric inference (2005 Stata Statistical Software Release9; Stata Corp., College Station, Texas, USA) was used.

## 3. Results

**Figure 1:**
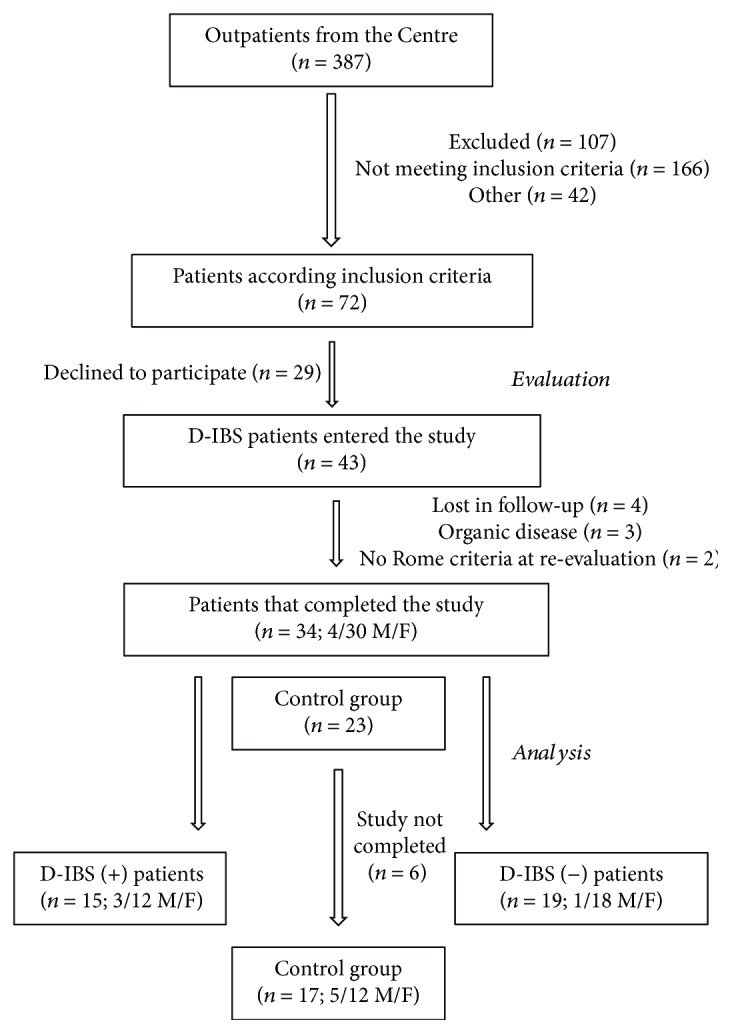
The flow of participants through the study.

Figure 1 shows the flow of participants through the study. Thirty-four D-IBS patients (4 men and 30 women; mean age = 41.2 ± 2.1 yrs.; BMI = 25.1 ± 0.9) and 17 HC subjects (5 men and 12 women; mean age = 39.5 ± 2.9 yrs.; BMI = 24.3 ± 0.5) completed the study. Regarding the anthropometric data and gender, no significant differences were present among groups (data not shown).

Table 1 shows the symptom score calculated with GSRS as both the single symptoms and symptom clusters in HC, in the whole D-IBS group, and the two subgroups of D-IBS. As expected, the profile of the IBS group was significantly different from that of the HC. In particular, the GSRS items related to indigestion (abdominal distension and flatulence) were those with the highest median score. The comparison between the two D-IBS subgroups showed that D-IBS(−) patients had a significantly higher GSRS cluster pain and diarrhoea profile than the D-IBS(+).

Table 2 reports the correlations between the GSRS clusters and the haematological variables considering as significant (highlighted in bold) only those correlations that satisfied the Bonferroni correction (9 variables 0.05/9 = 0.005). As one can see, there were significant and marked correlations between the symptoms clusters and the immune activation and intestinal inflammation markers, as well as BDNF.

All the D-IBS patients and HC subjects underwent intestinal permeability testing. As for %Ma, D-IBS patients had significantly lower (*p* = 0.0386) urinary excretion values than HC (11.03 ± 0.51 *vs* 12.98 ± 0.58). On the contrary, %La was higher in patients than controls, although without reaching a significant difference (0.409 ± 0.055 *vs* 0.237 ± 0.029; *p* = 0.1212). Consequently, the La/Ma ratio was significantly higher (*p* = 0.0091) in D-IBS than HC (0.0367 ± 0.005 *vs* 0.0186 ± 0.002).

Fifteen D-IBS patients with a La/Ma ratio equal to or higher than 0.035 were categorised as D-IBS(+), and 19 with a La/Ma ratio lower than 0.035 as D-IBS(−).

To evaluate whether the immune system was activated in D-IBS patients and such activation could be associated with an impaired epithelial barrier, plasma concentrations of proinflammatory adipo(cyto)kines were determined in HC and D-IBS patients with normal and increased intestinal permeability.

**Figure 2:**
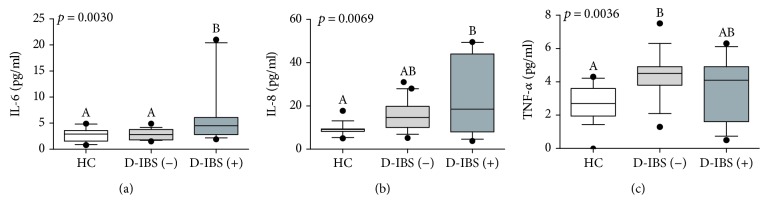
The plasma levels of IL-6, IL-8, and TNF-*α* in healthy controls (HC) and diarrhoea-predominant IBS (D-IBS) patients categorised as having normal or increased s-IP. D-IBS patients with a lactulose to mannitol ratio equal to or higher than 0.035 were categorised as D-IBS(+) and patients with a ratio value lower than 0.035 as D-IBS(−). Data are reported as box and whiskers representing 10–90 percentile. Kruskal-Wallis test and Dunn's multiple comparison test were used for the statistical analysis. Box and whiskers not showing a common letter differ significantly (*p* < 0.05).

Figure 2 reports the plasma levels of IL-6, IL-8, and TNF-*α* in HC and patients categorised as having normal or increased s-IP. Statistically significant differences in the plasma concentrations of all the adipo(cyto)kines were found among the groups. IL-6 levels were significantly different among D-IBS(+), D-IBS(−), and HC (*p* = 0.0030). Besides, D-IBS(+) patients showed the highest IL-6 levels compared to both D-IBS(−) and HC, statistically significant at the Dunn's post hoc test (*p* < 0.05) (Figure 2(a)). The plasma IL-8 levels were significantly different among the three groups (*p* = 0.0069), and the circulating concentrations of this cytokine were significantly higher in D-IBS(+) compared to HC patients at the post hoc test (Figure 2(b)). Lastly, TNF-*α* levels differed among the groups (*p* = 0.0036), and D-IBS(−) patients showed significantly higher levels compared with HC but not with D-IBS(+) patients (*p* < 0.05; Figure 2(c)).

**Figure 3:**
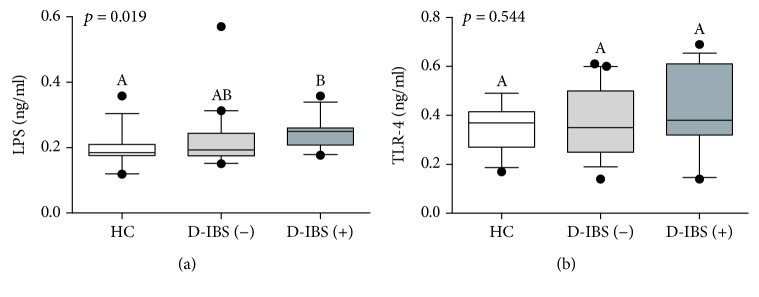
The plasma levels of LPS and TLR-4 in healthy controls (HC) and diarrhoea-predominant IBS (D-IBS) patients categorised as having normal or increased s-IP. D-IBS patients with a lactulose to mannitol ratio equal to or higher than 0.035 were categorised as D-IBS(+) and patients with a ratio value lower than 0.035 as D-IBS(−). Data are reported as box and whiskers representing 10–90 percentile. Kruskal-Wallis test and Dunn's multiple comparison test were used for the statistical analysis. Box and whiskers not showing a common letter differ significantly (*p* < 0.05).

Figure 3 reports the plasma levels of LPS and TLR-4. As for LPS concentrations, the statistical analysis revealed significant differences among groups (*p* = 0.019) and D-IBS(+) had significantly higher (*p* < 0.05) concentrations than HC at the post hoc test (Figure 3(a)). Finally, TLR-4 did not show significant differences among groups (*p* = 0.544; Figure 3(b)). A statistically significant correlation between LPS and IL-6 concentrations was found (*r* = 0.3119; *p* = 0.0259).

**Figure 4:**
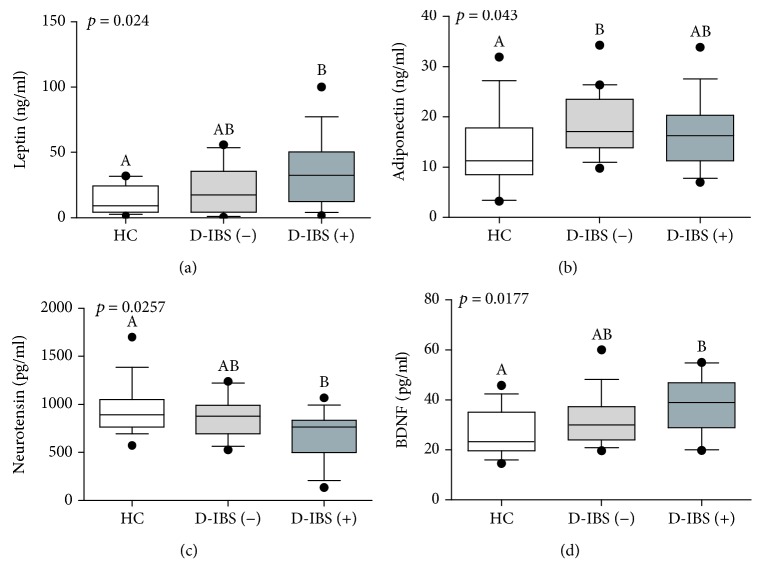
The circulating levels of leptin, adiponectin, BDNF, and neurotensin in healthy controls (HC) and diarrhoea-predominant IBS (D-IBS) patients categorised as having normal or increased s-IP. D-IBS patients with a lactulose to mannitol ratio equal to or higher than 0.035 were categorised as D-IBS(+) and patients with a ratio value lower than 0.035 as D-IBS(−). Data are reported as box and whiskers representing 10–90 percentile. Kruskal-Wallis test and Dunn's multiple comparison test were used for the statistical analysis. Box and whiskers not showing a common letter differ significantly (*p* < 0.05).

The serum levels of leptin, adiponectin, neurotensin, and BDNF in HC and D-IBS patients categorised as having normal or increased s-IP are reported in Figure 4. As for leptin, the difference among groups was significant (*p* = 0.024), and D-IBS(+) patients had significantly (*p* < 0.05) higher circulating levels than HC (Figure 4(a)). Also, adiponectin levels differed significantly among the groups (*p* = 0.043) and both the D-IBS groups showed higher adiponectin levels than HC, but the statistical difference at the post-test was found only between D-IBS(−) and HC (Figure 4(b)). The serum neurotensin levels were significantly different among groups (*p* = 0.0257), and D-IBS(+) patients showed significantly (*p* < 0.05) lower neurotensin levels compared to healthy controls at the post hoc test (Figure 4(c)). Finally, BDNF differed among the groups (*p* = 0.0177), and D-IBS(+) patients had significantly (*p* < 0.05) higher circulating BDNF levels than HC at the post hoc test (Figure 4(d)). Weak although statistically significant correlations were found between BDNF and IL-6 (*r* = 0.2792; *p* = 0.0473) and LPS levels (*r* = 0.2877; *p* = 0.0406).

To investigate on the possible genetic component in the regulation of the intestinal barrier functions, we evaluated the frequency distribution of some polymorphisms at the loci coding for the peptides under investigation and leptin receptor and compared them among HC and D-IBS patients with normal or increased intestinal permeability. The frequencies of genotypes were in agreement with Hardy-Weinberg equilibrium at all the analysed loci. No significant differences were in the frequency distribution of genotypes among the three study groups in none of the investigated SNPs when all the possible models of inheritance (recessive, dominant, and codominant) were considered. The same was for the allelic frequencies (see the “Supplementary material” section).

## 4. Discussion

In the present study, we categorised D-IBS patients as having normal or not normal s-IP, according to the levels of La and Ma secreted in the urine as biomarkers of the integrity of the GI barrier, and evaluated the symptom profiles and the adipose tissue-derived inflammatory and neuroendocrine factors in comparison with those in healthy subjects.

According to our results, 44% of D-IBS patients showed increased s-IP, and this finding suggests that alterations of the GI barrier function and integrity may not always be present in IBS, even in its diarrhoea variant. Our data are suggestive for at least two distinct subsets of D-IBS patients, and the investigation of possible s-IP alterations (i.e., considering the La/Ma ratio) might be useful to better evaluate this heterogeneous symptom-based syndrome.

Once more, it is evident that the analysis of symptoms may not be sufficient to discriminate patients with IBS. On the one hand, the D-IBS(−) patients showed single GSRS items perfectly overlapping those of D-IBS(+) patients. On the other, the comparison of clusters of GSRS highlighted a distinct symptom profile for D-IBS(−) patients, with higher scores for abdominal pain and diarrhoea than D-IBS(+) patients, thus supporting the existence of a lower pain threshold in the first group of patients [24]. Furthermore, a close correlation between the cluster scores of the GSRS items and some markers of inflammation (namely, IL-8, TNF-*α*, adiponectin, and leptin) along with BDNF was found. Available reports in the literature are contrasting. Recently, some authors tried to correlate the circulating levels of LPS, the LPS coreceptor soluble cluster of differentiation (sCD), monocyte chemoattractant protein 1 (MCP 1), intestinal fatty acid binding protein (IFABP), and calprotectin with the symptom scores calculated by the IBS Severity Scoring System, but they failed in identifying any associations [25]. In fact, in agreement with this report, we found no correlation between LPS and symptoms. By opposite, we found positive correlations between TNF-*α* and IL-8 and the clusters of symptoms related to abdominal pain and diarrhoea like other authors who found that these inflammation markers were significantly associated with the symptom scores of D-IBS patients [26]. These divergent results may depend on the differences in the adopted symptom questionnaires as well as the evaluated markers of inflammation.

Leptin and BDNF correlated with the indigestion cluster, while adiponectin correlated with the abdominal pain cluster. As concerns leptin, adiponectin, and BDNF, there are no data on the possible correlation between these molecules and IBS symptoms. However, all these molecules are in various degree involved in the inflammation processes of the GI tract, and their significant association with the pain and indigestion clusters found here is quite conceivable.

Variations in the circulating cytokines have been well demonstrated in IBS, although with some inconsistencies among the available studies [27]. The recognised heterogeneity among patients could represent a possible bias when determining the relevance of the immune system in the pathogenesis of IBS. Thus, there is the need for new strategies for IBS classification and diagnosis, overcoming/combining the symptom-based diagnosis with the use of new biohumoral markers that can help clinicians in its management [28].

A low-grade inflammation component is a relevant issue in the pathophysiology of IBS [1, 9]. Nevertheless, the aetiological role of immune-mediated events is difficult to define due to a shared psychoneuroimmunological pathway between immune system activation and stress, with the latter recognised to as a critical factor for the development of the syndrome [29]. An interplay between visceral adipose tissue and intestinal permeability has already been demonstrated [13]. Specifically, the increased intestinal permeability and consequent barrier dysfunction allow permeation of pathogenic microorganisms and their products. All these factors could drive the expression of mediators of inflammation in adipocytes, resulting in immune activation [30].

Our results about circulating proinflammatory adipo(cyto)kines depict an increased immune activation status in D-IBS patients, as shown by the higher levels of IL-6, IL-8, and TNF-*α* compared to HC. Besides, these data confirm the possible role of circulating cytokines as useful biomarkers in D-IBS [31], although this seems to be particularly true for the D-IBS patients with s-IP alteration. Previous results on the association between increased proinflammatory cytokines and clinical symptoms mainly addressed psychological factors [32], whereas our study was focused on a possible association between the integrity of the mucosal barrier and immune activation. The levels of these mediators of inflammation in D-IBS(+) patients were higher than those of controls, and IL-6 levels correlated with those of LPS. This evidence is in line with the assumption that the activation and maintenance of the low-grade mucosal inflammation in IBS relies on an impaired intestinal barrier leading to increased endotoxemia [6, 7].

In the adipose tissue, TNF-*α* acts as a secretagogue of leptin, an adipokine that links nutrition with immunity [33]. No definitive role for this peptide has been established in the course of D-IBS. Anty et al. [34] found significantly higher circulating levels of leptin compared with HC in D-IBS patients, while Semnani et al. [35] found reduced levels of protein in patients compared to healthy subjects and explained this reduction by relating it to stress. In our study, D-IBS(+) patients showed values of leptin significantly higher than those of controls. These higher circulating levels could be explained by taking into consideration the direct role of leptin as a critical mediator of intestinal permeability through the activation of signalling pathways regulating cell metabolism and tight junction integrity [36]. It has been demonstrated that this peptide can increase tight junction permeability, independently of inflammation status [37]. Besides, since a proinflammatory role contributing to barrier damage has been demonstrated for colonic leptin in IBD [38], the increased circulating levels of this hormone in our D-IBS(+) patients could also reflect the inflammatory processes occurring in the gut.

As regards adiponectin, the levels of this hormone were higher in our D-IBS patients than in HC. Although there is general agreement on the role of adiponectin as a local mediator impacting on the intestine, its exact pro/anti-inflammatory effect remains obscure, as reviewed by Karrasch and Schaeffler [39]. The increased levels of adiponectin we found were consistent with a possible proinflammatory role of this molecule in IBS or may be representative of a counter response to the immune activation, as already demonstrated in patients suffering from IBD [40]. Adiponectin values, however, were almost similar in D-IBS(+) and D-IBS(−) patients, confirming the absence of a role for this hormone in the maintenance of intestinal permeability [37].

We evaluated the circulating levels of the neuropeptide neurotensin and the metabotropic BDNF, which are neuroendocrine factors expressed in adipose tissue possibly involved in modulating inflammation and mucosal barrier integrity. Neurotensin acts as a neurotransmitter in the CNS and the ENS [41] and as a hormone peripherally. Through paracrine and endocrine mechanisms, it regulates GI secretion and motility [42]. Additionally, neurotensin plays many roles in GI disorders, and all of its properties still have not been clarified. Not only was it shown as being implicated in intestinal inflammation and dysmetabolic conditions, as demonstrated by its increased peripheral levels found in obese patients [43], but it also has been considered as an active trophic factor for the GI tract, since it has been shown to improve intestinal barrier function [44]. This evidence leads to interesting pathophysiological implications for barrier integrity in D-IBS, and the significantly lower levels of the neuropeptide in our patients with altered s-IP compared to HC agree with this proposed role.

Conversely, BDNF was significantly higher in patients with increased permeability than in controls. According to its functional pleiotropy, altered expression of BDNF has been associated with different comorbidities of chronic GI diseases. Increased expression of BDNF in colonic mucosa has been found in biopsies from IBS patients [10], and more recently, the role of BDNF in colon hypersensitivity and its mechanism have been demonstrated in rats [45]. Besides, a specific function for colonic BDNF in modulating intestinal barrier integrity has been described [10]. Neurotrophin also represents a possible link between the nervous and immune systems: BDNF is thought to have a crucial role in modulating neuroinflammation by promoting neuroprotection, because high levels can increase neuronal resistance to metabolic stress [46]. Our data on BDNF, which was significantly higher in D-IBS(+) patients than in controls along with its significant correlations with LPS and IL-6, are in agreement with this scenario.

A growing appreciation of the role of genetics on the pathophysiology of IBS exists, in particular, on the possible influence of polymorphisms of some cytokines and neuropeptides [47]. The multifactorial nature of this symptom-based disorder presents a big challenge for the characterization of IBS-related genes [20]. As a working hypothesis, we searched for an association between genetic variants in genes coding for the proteins we have investigated together with the gene for the leptin receptor and alterations of intestinal permeability. Unfortunately, none of the analysed SNPs showed a significantly different frequency distribution between patients and healthy subjects. However, one limit of the study was the relatively small number of studied subjects that prevents us from drawing firm conclusions.

## 5. Conclusions

The pathophysiology of IBS is complex, and our findings could be of some help in the characterization of the D-IBS variant, although they need verification in a larger cohort of patients. The present results highlight the contribution of an altered intestinal barrier in the pathogenesis of this syndrome, at least of this specific subgroup of IBS. In this framework, a role could be ascribed to molecules secreted by the visceral adipose tissue that can impact on barrier functions. It would be interesting to evaluate whether the molecules investigated here are predictive for patients' outcome/response to treatment, as already reported by other groups [31]. Overall, the results from this paper are encouraging, and they warrant further study aimed to better characterise their role in IBS pathogenesis and to allow future targeted therapies for specific IBS subgroups.

## Figures and Tables

**Table 1:** Comparison of GSRS score between HC and D-IBS and between D-IBS (−) and D-IBS (+) patients.

	HC	D-IBS	D-IBS (−)	D-IBS (+)
*GSRS single items*				
Epigastric pain	1 (1–1)	3.5 (1–7)^∗^	5 (1–7)	2 (1–6)
Gastric hunger pain	1 (1–1)	2 (1–7)^∗^	1 (1–7)	2 (1–5)
Abdominal continuous pain	1 (1–1)	1 (1–5)^∗^	3 (1–5)	1 (1–5)
Abdominal colic pain	1 (1–1)	2 (1–7)^∗^	5 (1–7)	1 (1–7)
Abdominal indefinite pain	1 (1–1)	1 (1–5)	1 (1–5)	1 (1–3)
Nausea	1 (1–1)	1 (1–6)^∗^	2 (1–6)	1 (1–4)
Burping	1 (1–1)	2 (1–7)^∗^	1 (1–7)	3 (1–5)
Borborygmi	1 (1–1)	2 (1–7)^∗^	2 (1–7)	2 (1–7)
Abdominal distension	1 (1–1)	5 (1–7)^∗^	5 (1–7)	5 (1–7)
Flatulence	1 (1–1)	4.5 (1–7)^∗^	5 (1–7)	3 (1–6)
Postprandial fullness	1 (1–1)	3 (1–7)^∗^	4 (1–7)	3 (1–5)
Early satiety	1 (1–1)	1 (1–7)^∗^	1 (1–7)	1 (1–7)
Increased passage of stools	1 (1–1)	1 (1–7)^∗^	1 (1–3)	1 (1–7)
Soft stool	1 (1–1)	1 (1–7)^∗^	3 (1–5)	1 (1–7)
Urgent bowel movement	1 (1–1)	1.5 (1–7)^∗^	1 (1–7)	2 (1–7)
Bristol score	4 (3–4)	4 (3–7)^∗^	5 (3–6)	3 (3–7)
*GSRS cluster scores*				
Pain syndrome	6 (6–6)	13 (6–25)^∗^	16 (7–25)	11 (6–22)^∗^
Indigestion syndrome	6 (6–6)	19 (7–38)^∗^	19 (7–38)	22 (12–36)
Diarrhoea syndrome	3 (3–3)	5 (3–21)^∗^	7 (3–15)	5 (3–21)^∗^

HC: healthy controls; D-IBS (+)/(−): diarrhoea-predominant IBS according to presence/absence of impaired permeability. Data are expressed as median and range and were analysed by rank sum test. ^∗^(*p* < 0.05).

**Table 2:** Correlation between GSRS combination score and analytical measurements in all subjects (51 subjects; HC, D-IBS (−), and D-IBS (+) patients).

	Abdominal pain cluster [r (p)]	Indigestion cluster [r (p)]	Diarrhoea cluster [r (p)]
*IL6*	0.134 (0.347)	0.118 (0.409)	0.183 (0.198)
*IL8*	0.442 **(0.00126)**	0.361 (0.00941)	0.428 **(0.00182)**
*TNF-alfa*	0.434 **(0.00157)**	0.354 (0.0110)	0.421 **(0.00224)**
*LPS*	0.0259 (0.856)	0.178 (0.211)	0.096 (0.517)
*TLR-4*	−0.0801 (0.575)	−0.0586 (0.682)	0.0978 (0.493)
*Leptin*	0.210 (0.139)	0.452 **(0.000943)**	0.0828 (0.562)
*Adiponectin*	0.428 **(0.00187)**	0.293 (0.0368)	0.303 (0.0309)
*BDNF*	0.353 (0.0113)	0.404 **(0.00348)**	0.0668 (0.640)
*NT*	−0.0787 (0.581)	−0.159 (0.263)	−0.188 (0.184)

HC: healthy controls; D-IBS (+)/(−): diarrhoea-predominant IBS according to presence/absence of impaired permeability. Analysis was performed by the Spearman correlation coefficient. The correlations that satisfied the Bonferroni correction (9 variables 0.05/9 = 0.005) are highlighted in bold.

## Data Availability

The datasets used and/or analysed during the current study are available from the corresponding author on reasonable request.

## Supplementary Materials

Supplementary Materials The supplementary material section contains two tables. Tables 3 reports the primer sequence and reaction conditions for genotyping assays along with a list of references. Table 4 reports the list of genetic polymorphisms in the study groups.
