# Supplementary material for: Adipose Tissue-Derived Biomarkers of Intestinal Barrier Functions for the Characterization of Diarrhoea-Predominant IBS
Source: Dis Markers. 2018 Nov 28;2018:1827937. doi: 10.1155/2018/1827937 (PMC6304194; doi:10.1155/2018/1827937)
Supplement: Supplementary Materials — The supplementary material section contains two tables. Tables 3 reports the primer sequence and reaction conditions for genotyping assays along with a list of references. Table 4 reports the list of genetic polymorphisms in the study groups. [file 1827937.f1.docx]

**Table 3**. Primers sequence and reaction conditions for genotyping assays

| **Polymorphism** | **Primer sequences** | **Annealing**  **Temperature**  **(°C)** | **Restriction**  **enzyme** | **Informative fragments length (bp)** | **Ref.** |
| --- | --- | --- | --- | --- | --- |
| ***IL-6 -174 G/C (rs1800795*)** | F- 5’CACTCCACCTGGAGACGCCT3′  R- 5′TCCCTCACACAGGGCTCGAC3′ | 61 | NlaIII | 432  310+122 | [^[[1]](#endnote-1)^] |
|  |  |  |  |  |  |
| ***IL8-251 A/T (rs4073)*** | F- 5’CCATCATGATAGCATCTGT3’  R- 5’CCACAATTTGGTGAATTATTAA3’ | 57 | AseI | 173  152+21 | [^[[2]](#endnote-2)^] |
|  |  |  |  |  |  |
| ***IL8 +781 C/T (rs2227306)*** | F- 5’CTCTAACTCTTTATATAGGAATT3’  R- 5’GATTGATTTTATCAACAGGCA3’ | 52 | EcoRI | 203  184+19 | [^[[3]](#endnote-3)^] |
|  |  |  |  |  |  |
| ***TNFα-308 G/A (rs1800629)*** | F-5’AGGCAATAGGTTTTGAGGGCCAT3’  R-5’GAGCGTCTGTGGCTGGGTG3’ | 58 | NcoI | 345  325+20 | [^[[4]](#endnote-4)^] |
|  |  |  |  |  |  |
| ***LEP G2548A (rs7799039)*** | F- 5’TTTCCTGTAATTTTCCCATGAG3’  R- 5’AAAGCAAAGACAGGCATAAAA3’ | 53 | HhaI | 242  181+61 | [^[[5]](#endnote-5)^] |
|  |  |  |  |  |  |
| ***LEPR Q223R (rs1137101)*** | F- 5’ACCCTTTAAGCTGGGTGTCCCAAATGA3’  R- 5’ CTAGCAAATATTTTTGTAAGCAATT3 | 55 | MspI | 440  300+140 | [^[[6]](#endnote-6)^] |
|  |  |  |  |  |  |
| ***ADIPOQ G276T (rs1501299)*** | F- 5′CCTGGTGAGAAGGGTGAGAA3’  R- 5′AGATGCAGCAAAGCCAAAGT3 | 58 | BsmI | 241  148+93 | [^[[7]](#endnote-7)^] |
|  |  |  |  |  |  |
| ***NTS G479A (rs1800832)*** | F-5’GCTGAAGGAAAGAGGAAGTG3’  R-5’GGAGTAGCATGCATACAAGC3’ | 55 | DdeI | 128  79+49 | [^[[8]](#endnote-8)^] |
|  |  |  |  |  |  |
| ***BDNF G196A (rs6265)*** | F-5′ACTCTGGAGAGCGTGAAT3’  R-5′ATACTGTCACACACGCTC3’ | 58 | NlaIII | 243  75+168 | [^[[9]](#endnote-9)^] |

**Table 4.** Genetic polymorphisms in the study groups.

| **Polymorphism** | **HC n (%)** | **D-IBS (-) n (%)** | **D-IBS(+) n (%)** | **Statistics** | **p** |
| --- | --- | --- | --- | --- | --- |
| ***IL-6 -174 G/C (rs1800795*)** |  |  |  |  |  |
| **GG** | 10 (59) | 11 (58) | 10 (67) | Recessive CC vs. GC+GG | 0.5355 |
| **GC** | 6 (35) | 8 (42) | 4 (27) | Dominant GG vs. GC+CC | 0.8557 |
| **CC** | 1 (6) | 0 (0) | 1 (7) | Codominant GC vs. GG | 0.7257 |
| **G** | 26 (76) | 30 (79) | 24 (80) | CC vs. GG | 0.5873 |
| **C** | 8 (24) | 8 (21) | 6 (20) | Allelic C vs. G | 0.9385 |
| **HWE p=0.757793** |  |  |  |  |  |
|  |  |  |  |  |  |
| ***IL8-251 A/T (rs4073)*** |  |  |  |  |  |
| **TT** | 6 (35) | 9 (47) | 3 (20) | Recessive AA vs. TA+TT | 0.7537 |
| **TA** | 7 (41) | 6 (32) | 10 (67) | Dominant TT vs. TA+AA | 0.2529 |
| **AA** | 4 (24) | 4 (21) | 2 (13) | Codominant TA vs. TT | 0.1427 |
| **T** | 19 (56) | 24 (63) | 16 (53) | AA vs. TT | 0.8788 |
| **A** | 15 (44) | 14 (37) | 14 (47) | Allelic A vs. T | 0.6894 |
| **HWE p=0.590820** |  |  |  |  |  |
|  |  |  |  |  |  |
| ***IL8 +781 C/T (rs2227306)*** |  |  |  |  |  |
| **CC** | 8 (47) | 10 (53) | 6 (40) | Recessive TT vs. CT+CC | 0.6312 |
| **CT** | 6 (35) | 6 (31) | 8 (53) | Dominant CC vs. CT+TT | 0.7646 |
| **TT** | 3 (18) | 3 (16) | 1 (7) | Codominant CT vs. CC | 0.5439 |
| **C** | 22 (65) | 26 (68) | 20 (67) | TT vs. CC | 0.8123 |
| **T** | 12 (35) | 12 (32) | 10 (33) | Allelic T vs. C | 0.9458 |
| **HWE p=0.400814** |  |  |  |  |  |
|  |  |  |  |  |  |
| ***TNFα-308 G/A (rs1800629)*** |  |  |  |  |  |
| **GG** | 13 (76) | 17 (89) | 12 (80) | Recessive AA vs. GA+GG | nd |
| **GA** | 4 (24) | 2 (11) | 3 (20) | Dominant GG vs. GA+AA | nd |
| **AA** | 0 (0) | 0 (0) | 0 (0) | Codominant GA vs. GG | 0.5698 |
| **G** | 30 (88) | 36 (95) | 27 (90) | AA vs. GG | nd |
| **A** | 4 (12) | 2 (5) | 3 (10) | Allelic A vs. G | 0.6017 |
| **HWE p=0.489499** |  |  |  |  |  |
|  |  |  |  |  |  |
| ***LEP G2548A (rs7799039)*** |  |  |  |  |  |
| ***GG*** | 5 | 4 | 5 | Recessive AA vs. GA+GG | 0.5591 |
| **GA** | 10 | 11 | 6 | Dominant GG vs. GA+AA | 0.7103 |
| **AA** | 2 | 4 | 4 | Codominant GA vs. GG | 0.6056 |
| **G** | 20 | 19 | 16 | AA vs. GG | 0.6869 |
| **A** | 14 | 19 | 14 | Allelic A vs. G | 0.7527 |
| **HWE p=0.640581** |  |  |  |  |  |
|  |  |  |  |  |  |
| ***LEPR Q223R (rs1137101)*** |  |  |  |  |  |
| ***AA*** | 8 (47) | 7 (37) | 8 (53) | Recessive GG vs. AG+AA | 0.8816 |
| ***AG*** | 7 (41) | 10 (53) | 6 (40) | Dominant AA vs. AG+GG | 0.6187 |
| ***GG*** | 2 (12) | 2 (10) | 1 (7) | Codominant AG vs. AA | 0.6435 |
| **A** | 23 (68) | 24 (63) | 22 (73) | GG vs. AA | 0.5901 |
| **G** | 11 (32) | 14 (37) | 8 (27) | Allelic G vs. A | 0.6727 |
| **HWE p=0.828671** |  |  |  |  |  |
|  |  |  |  |  |  |
| ***ADIPOQ G276T (rs1501299)*** |  |  |  |  |  |
| ***GG*** | 7 (41) | 12 (63) | 8 (53) | Recessive TT vs. GT+GG | 0.7718 |
| ***GT*** | 9 (53) | 5 (26) | 5 (33) | Dominant GG vs. GT+TT | 0.4187 |
| ***TT*** | 1 (6) | 2 (11) | 2 (13) | Codominant GT vs. GG | 0.2852 |
| ***G*** | 23 (68) | 29 (76) | 21 (70) | TT vs. GG | 0.8943 |
| ***T*** | 11 (32) | 9 (24) | 9 (30) | Allelic T vs. G | 0.6997 |
| **HWE p=0.545954** |  |  |  |  |  |
|  |  |  |  |  |  |
| ***NTS G479A (rs1800832)*** |  |  |  |  |  |
| ***AA*** | 12 (71) | 15 (79) | 11 (73) | Recessive GG vs. AG+AA | 0.2941 |
| ***AG*** | 5 (29) | 4 (21) | 3 (20) | Dominant AA vs. AG+GG | 0.8413 |
| ***GG*** | 0 (0) | 0 (0) | 1 (7) | Codominant AG vs. AA | 0.8129 |
| ***A*** | 29 (85) | 34 (89) | 25 (83) | GG vs. AA | 0.3152 |
| ***G*** | 5 (15) | 4 (11) | 5 (17) | Allelic G vs. A | 0.7501 |
| **HWE p= 0.963013** |  |  |  |  |  |
|  |  |  |  |  |  |
| ***BDNF G196A (rs6265)*** |  |  |  |  |  |
| ***GG*** | 10 (59) | 12 (63) | 10 (67) | Recessive AA vs. GA+GG | 0.4236 |
| ***GA*** | 7 (41) | 6 (32) | 5 (33) | Dominant GG vs. GA+AA | 0.8995 |
| ***AA*** | 0 (0) | 1 (5) | 0 (0) | Codominant GA vs. GG | 0.8609 |
| ***G*** | 27 (79) | 30 (79) | 25 (83) | AA vs. GG | 0.4524 |
| ***A*** | 7 (21) | 8 (21) | 5 (17) | Allelic A vs. G | 0.8888 |
| **HWE p=0.393389** |  |  |  |  |  |

1. Vickers MA, Green FR, Terry C, Mayosi BM, Julier C, Lathrop M et al. Genotype at a promoter polymorphism of the interleukin-6 gene is associated with baseline levels of plasma C-reactive protein. Cardiovascular Res 2002; 53: 1029–1034 [↑](#endnote-ref-1)
2. Xu D, Li J, Huang X, Lin A, Gai Z, Chen Z. Impact of IL-8-251A/T gene polymorphism on severity of disease caused by enterovirus 71 infection. Arch Virol. 2016 Jan;161(1):203-7. doi: 10.1007/s00705-015-2645-2. [↑](#endnote-ref-2)
3. Li JA, Chen ZB, Lv TG, Han ZL. Genetic polymorphism of CCL2-2518, CXCL10-201, IL8+781 and susceptibility to severity of Enterovirus-71 infection in a Chinese population. Inflamm Res. 2014 Jul;63(7):549-56. doi: 10.1007/s00011-014-0724-6. [↑](#endnote-ref-3)
4. Sakao S, Tatsumi K, Igari H, Watanabe R, Shino Y, Shirasawa H, Kuriyama T. Association of tumor necrosis factor-alpha gene promoter polymorphism with low attenuation areas on high-resolution CT in patients with COPD. Chest. 2002 Aug;122(2):416-20. [↑](#endnote-ref-4)
5. Amer T, El-Baz R, Mokhtar AR, El-Shaer S, Elshazli R, Settin A. Genetic polymorphisms of IL-23R (rs7517847) and LEP (rs7799039) among Egyptian patients with hepatocellular carcinoma. Arch Physiol Biochem. 2017 Dec;123(5):279-285. doi: 10.1080/13813455.2017.1320680. [↑](#endnote-ref-5)
6. El-Hussiny MA, Atwa MA, Rashad WE, Shaheen DA, Elkady NM. Leptin receptor Q223R polymorphism in Egyptian female patients with breast cancer. Contemp Oncol (Pozn). 2017;21(1):42-47. doi: 10.5114/wo.2017.66655. [↑](#endnote-ref-6)
7. Sandhu HS, Puri S, Sharma R, Sokhi J, Singh G, Matharoo K, Bhanwer A. Associating genetic variation at Perilipin 1, Complement Factor D and Adiponectin loci to the bone health status in North Indian population. Gene. 2017 Apr 30;610:80-89. doi: 10.1016/j.gene.2017.02.009. [↑](#endnote-ref-7)
8. J. Vanakoski, C. Mazzanti, H. Naukkarinen, M. Virkkunen, andD.Goldman, “An abundant proneurotensin polymorphism, 479A>G, and a test of its association with alcohol dependence in a finnish population,” Alcoholism: Clinical and Experimental Research, vol. 24, no. 6, pp. 762–765, 2000. [↑](#endnote-ref-8)
9. Chen S-P, Fuh J-L, Wang S-J, Tsai S-J, Hong C-J, Yang AC (2011) Brain-derived neurotrophic factor gene Val66Met polymorphism modulates reversible cerebral vasoconstriction syndromes. PLoS ONE 6:e18024 [↑](#endnote-ref-9)
